# Unusual Clinical and Pathological Features in Pemphigus Vulgaris: A Potential Diagnostic Pitfall 

**DOI:** 10.1155/2011/518758

**Published:** 2011-03-16

**Authors:** Rosario Caltabiano, Gaetano Magro, Lidia Puzzo, Enrico Vasquez, Rocco De Pasquale

**Affiliations:** ^1^Section of Anatomic Pathology, Department G.F. Ingrassia, University of Catania, 95123 Catania, Italy; ^2^Department of Dermatology, University of Catania, “G. Rodolico” Polyclinic, 95123 Catania, Italy

## Abstract

We describe the case of a 67-year-old woman affected by pemphigus vulgaris with a dry whitish scaly lesion in the upper lip. Clinically, this lesion resembled an actinic keratosis. Although histological examination revealed a focal acantholysis, the finding of a moderate-to-severe dysplastic epithelium was consistent with the diagnosis of acantholytic actinic keratosis with moderate/severe dysplasia. Nevertheless, the complete resolution of the lip lesion after systemic therapy for pemphigus vulgaris led us to reconsider the possibility that we were dealing with a pemphigus vulgaris with unusual clinical and histological features. The previously reported cytological dysplasia was better regarded reactive rather than neoplastic, likely as the result to the inflammatory injury.

## 1. Introduction

**Figure 1:**
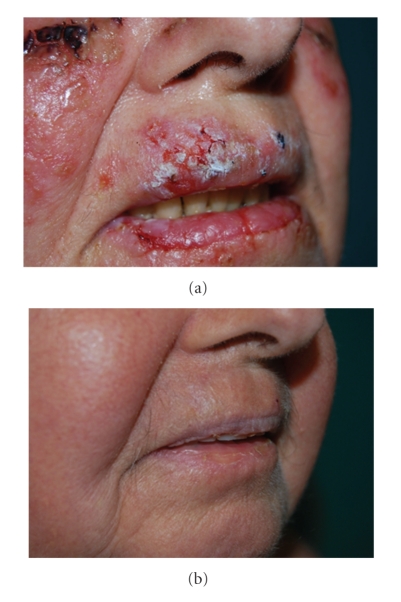
(a) Dry whitish scaly lesion of the upper lip, clinically suspicious for actinic keratosis. (b) Complete clinical resolution of the upper lip lesion.

**Figure 2:**
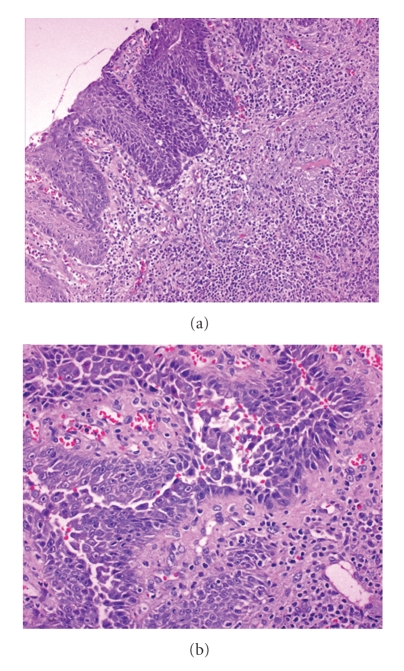
(a) The underlying dermis showed diffuse and marked mixed inflammatory infiltrate, consisting of lymphocytes and eosinophils, which focally involved the underlying epidermis. Additionally solar elastosis and vascular ectasia were seen (H & E; 100x). (b) Acantholysis and a diffuse cytological atypia of the epidermis, consisting of cells with enlarged, vesicular, focally hyperchromatic, nuclei (H & E; 200x).

A 67-year-old woman was referred to our department with a 4-month history lesions on her face, trunk, and upper extremities. The lesions consisted of multiple painful blisters and erosions covered with crusts. General laboratory screening, including a complete blood count, was within normal limits. The Tzanck test of a smear from the base of a blister on a trunk lesion showed many acantholytic cells. The indirect immunofluorescence test showed that the serum of the patient contained IgG antibodies that reacted with the intercellular region of normal squamous epithelium. The direct immunofluorescence test, performed in a cutaneous biopsy, showed IgG and C3 deposits to the full thickness of the epidermis. The immunoblotting revealed the presence of antibodies directed at the extracytoplasmic domain of the 130 kD epithelial desmosomal cadherin, desmoglein 3. Based on these findings, the diagnosis of pemphigus vulgaris was made. Unexpectedly, a dry whitish scaly lesion of the upper lip, that resembled clinically an actinic keratosis, was also observed (Figure 1(a)). Therefore, we decided to perform an incisional biopsy on this lesion. Histological examination showed acantholysis and a diffuse cytological atypia of the epidermis, consisting of cells with enlarged, vesicular, focally hyperchromatic, nuclei (Figures 2(a) and 2(b)). The underlying dermis showed diffuse and marked inflammatory infiltrate, with lymphocytes and eosinophils, that focally involved the epidermis. Solar elastosis and vascular ectasia were additionally seen (Figure 2(a)). Although the finding of acantholysis was consistent with the diagnosis of pemphigus vulgaris, the possibility of an acantholytic actinic keratosis with moderate/severe dysplasia could not be completely ruled out. We decided to undertake the therapy for pemphigus vulgaris and to wait for the outcome of the lesion of the lip. Therapy with systemic prednisone (50 mg/die), cyclophosphamide (25 mg/die), and erythromycin (1800 mg/die) was administered with good results. Blisters, erosions, and unexpectedly also the dry scaly lesion of the upper lip disappeared and no obvious relapse has been noted after a 4-month follow-up period (Figure 1(b)).

## 2. Discussion

Pemphigus vulgaris usually begins in the mouth with painful erosions, and after a period of weeks or months, the blisters spread to involve the skin [1, 2]. The mouth lesions, including those occurring in the lip, clinically present as fragile, flaccid blister, which develops on normal or erythematous skin/mucosa, and rapidly rupture, leaving a painful crusted, bloody erosion [2]. Histologically, these lesions are characterized by rounded acantholytic cells with intensely eosinophilic cytoplasm, pyknotic nuclei, and perinuclear halos. The most striking feature in our case was the finding of a dry whitish scaly lesion in the upper lip, that closely resembled of an actinic keratosis. Histologically, this lesion showed focal acantholytic epidermis with moderate-to-severe cytological atypia in association with a diffuse and marked dermal inflammatory infiltrate. However, the resolution of the lip lesion after systemic therapy for pemphigus vulgaris was strongly in favour of the diagnosis of pemphigus vulgaris with unusual clinical and histological features. In this regard, the detected cytological atypia was better regarded to be reactive rather than dysplastic, likely as the result of diffuse dermal inflammatory injury. Therefore, although benign and malignant tumours may occasionally arise in the context of some acantholytic diseases [3–5], the diagnosis of dysplasia/in situ carcinoma should be made with extreme caution in patients affected by pemphigus vulgaris. 

In conclusion, considering that the observation we made may be seen in many patients affected by pemphigus vulgaris, such patients would benefit more from the therapy for pemphigus vulgaris instead of a biopsy. This would save the patient the pain of the biopsy and also the lack of necessity for it.
